# Modern Developments of the Marvellous
**Histoire du Merveilleux, dans les Temps Modernes. *Par Louis Figuier. Paris, 1860.*The Spiritual Magazine. *London, 1860.*The Arcana of Christianity, and various Sermons. *By the Rev. T. L. Harris.


**Published:** 1860-10-01

**Authors:** 


### Art. VII.—MODERN DEVELOPMENTS OF THE MARVELLOUS.*

The pretensions of the persons who call themselves spiritualists, and who have been commonly known heretofore by the less dignified title of "spirit-rappers," have lately been advanced with a boldness, and pushed with a pertinacity, that seem to demand some examination from the press. As long as the performances at a spiritual *séance* were considered esoteric, and the delusions of the unfortunate mediomaniacs were displayed only within the charmed circle of their deranged disciples, it was possible to cherish a hope that this trans-Atlantic folly might gain no permanent footing upon our shores, and to believe that a total disregard of its existence would best promote the great blessing of its final consumma-

* *Histoire du Merveilleux, dans les Temps Modernes.* Par Louis Figuier. Paris, 1860.

*The Spiritual Magazine.* London, 1860.

*The Arcana of Christianity, and various Sermons.* By the Rev. T. L. Harris.

tion. The time for these opinions has gone by. Finding a congenial soil in the presumptuous ignorance of the half-educated classes, aided in its spread by the wonderful facilities for the dissemination of opinion that our day affords, appealing to desires and passions always powerful in the human breast, and actively promoted as the source of a profitable calling by many whose barefaced knaveries can be ascribed neither to credulity nor disease, spiritualism bids fair to become one of the institutions of the time. It is incumbent upon us, therefore, to devote a portion of our space to an inquiry into the origin and tendencies of this growing evil; and while our admirable friend *Punch*, ever watchful and right-minded, brings down his terrible baton upon the ridiculous aspects of the question, we, on our part, must strive to range the phenomena of modern spiritualism under their appropriate psychical laws, and to show the precise analogy between this and former epidemics of a kindred nature.

A somewhat similar task has been undertaken by M. Louis Figuier, in the work the title of which appears at the head of this article, and the scope and objects of which are thus described by the author.

"After casting a rapid glance (we translate freely from the preface) at the marvellous as exhibited in antiquity and in the Middle Ages—a necessary preparation for its study in modern times—the first volume contains the history of the demoniacs of Loudun, and of the Jansenist convulsionaries, showing the marvellous again reigning supreme in the domain of theology.

"In the second volume the history of the Protestant prophets presents the characteristic type of those epidemics of delirium, excited by religious exaltation, of which the history of medicine affords so many examples; and the account of the divining rod exhibits to us one of the most singular developments of the marvellous, and one for which it was most difficult to find a philosophical explanation.

"The two other volumes designed to complete the work will contain the history of animal magnetism, of table-turning, and of spirit-rapping.

"We intend that each of these recitals should be followed or accompanied by that natural explanation which can now be given of the pretended prodigies described. In most cases the lights afforded by physiology and medicine are sufficient for this purpose.

"We believe that these discussions will produce in the mind of the reader a perfect conviction of the non-existence of supernatural agents, and a certainty that all the marvels which at various times have excited the surprise or the admiration of mankind can be explained by a knowledge of our physiological organization.

"A denial of the marvellous is therefore the philosophical conclusion to be drawn from this work, which might indeed be called *the marvellous explained.* And if we succeed in carrying our readers with us to this end, we shall think that we render a very real service to society—both to those who enshroud themselves voluntarily in the dangerous shadow of the mysticism so unfortunately resuscitated in our time, and to those who halt between two opinions, wanting the evidence necessary to confirm them in convictions or to guide them in conduct."

Of these volumes the first three are already published; and in them the subject of animal magnetism is concluded, leaving only table-turning and spirit-rapping to be discussed. A brief abstract of M. Figuier's completed labours will be advantageous as an introduction to the statements of modern spiritualists; and we will therefore endeavour to place before our readers some account of the general scope and tenor of his work. The special narratives are ushered in by a general introduction, which commences as follows:—

"The phenomena of table-turning have been the signal, in both hemispheres, for the appearance of wonders which recall to mind, or, with little variation, even reproduce, the most surprising acts recorded in the histories of the magicians of antiquity. A study of the supernatural manifestations which have furnished matters of dispute during so many years ought to aid in the comprehension of many remarkable facts recorded in history; and which, received with many reservations, or even totally rejected by the criticism of the two last centuries, should now possess for us their interest and their value. But this value will be greatly enhanced if the study, well pursued, brings us to the conclusion that contemporary marvels, like the ancient ones they so much resemble, are all connected by a natural link; and that, being all referrible to the same cause, they may be explained the one by the other; or, in other words, that a single marvel, thoroughly comprehended, will furnish a key to all the rest. Such a conclusion as this, removing every idea of supernatural agency, would be a victory gained by science over the spirit of superstition, to the great advancement of human reason and dignity.

"We purpose to enter upon this study from the double point of view of the critic and the historian. We shall endeavour to prove that the pretended supernatural manifestations by which the present century has been, and is now again disturbed, are only the consequences, the continuation, the necessary and inevitable developments of the phenomena of the same kind which have been displayed in the centuries preceding our own; and that all of them find their explanation in the nature of the human mind.

"The marvellous is, however, an aliment so necessary to humanity, that among all nations and in all times mankind have exhibited the same desire to believe in the wonderful and to admit the supernatural. The imagination of the masses can only sympathize with that which is astonishing. The harmony of the phenomena of the world around the order of nature, the undeviating regularity with which her laws work out their own fulfilment, or, in a word, all that is truly admirable and wonderful in the universe, is unable to satisfy that passion for marvels which distinguishes the vulgar, and which Horace condemus as unphilosophical—finding in the maxim 'wonder not' the foundation of true wisdom."

M. Figuier passes on, concisely enough, but still at too great length for precise translation, to glance at the time, long gone by, when the personal intervention of Deity, either actually, or according to popular belief, governed the acts of the more primitive human societies: an intervention recorded in Scripture, and dimly indicated through the dense clouds of mythology. As this intervention was actually, or was supposed to be, withdrawn, the priesthood gradually succeeded to some share of the authority once exercised by the Divinity—and were expected to exhibit, in some unmistakeable manner, signs of a Divine influence operating through them. Hence arose a demand on the part of the public—a necessity as regarded the priesthood—for some supernatural countersign in favour of every marked change of polity or laws, and such countersigns, according to differences of time and place, were called signs, miracles, or prodigies; and were renewed from time to time, either to subvert, or to support, the systems they had aided to establish.

The priests of Egypt, the Llamas, the Brahmins, the followers of Zoroaster, the Gnostics, the Pythagoreans, and the priestesses of the Delphian oracle, are enumerated as having among them practised all the arts, and taught all the essential doctrines of modern mediumship. To them we shall return hereafter.

Proceeding to the early Christian period, M. Figuier relates how the general inquietude of mind resulting from the conflict which raged between old and new opinions was especially favourable to manifestations of the marvellous; and how, moreover, these manifestations, which, for the most part, had formerly been limited to certain times and places, and fenced about by various mystic ordinances, then emancipated themselves from such restrictions. The temples and caves sacred to the pagan oracles became mute as the public faith departed from them—the sibyls deserted their ancient sanctuaries for an eternal exile—but oracles and sibyls found their successors in so-called magicians, whose performances were held beneath the open sky, and before the gaping crowds of towns and villages.

In the apostolic age there flourished two so-called magicians, Simon Magus and Apollonius Tyanaeus, whose skill and pretensions created no small discussion among the early Christians. "Many fathers of the Church," writes M. Figuier, "St. Justin among others, were not restrained from considering Simon as a god. The great magician had so mastered the faith both of Christians and of Pagans, that neither one nor the other dreamed of disputing the reality of his prodigies, but sought only to make the most of them. The Pagans regarded him as an emissary from the ancient gods, sent to manifest their power and to restore their waning influence. The Christians regarded him as operating by the immediate aid of demons, but still with the permission of their own true God. In all eyes, therefore, the performances of Simon were miraculous. When he made statues endued with powers of locomotion, that walked in the presence of the astonished and affrighted mob,—when he remained unhurt among the flames of a blazing pile,—when he changed stones to bread—all were miracles! and when, having raised himself from the ground in a chariot of fire by the aid of two demons, he fell, after a few seconds of elevation—a miracle again. For the Emperor Nero pronounced that this downfall ought not to be attributed to any natural cause, but to the victory of the God of the Christians; and the people, eagerly embracing the opinion that Cæsar had delivered as from certain knowledge, and still further imposed upon by his authority, declared with one voice that the fall of Simon was due to the prayer of St. Peter, which had destroyed the power of the demons of the magician.

In a note M. Figuier adds—

"The followers of Simon, whom the populace and even the senate of Rome worshipped as a god, raised a statue to him upon an island in the Tiber, with this inscription: *Simoni deo sancto.* Many fathers of the Church who read this inscription fully acknowledged the authenticity of Simon's miracles, and only protested against the attribute of *holiness* added to the divinity of the great magician. Others were not equally favourable to him, and alleged that he had sought to obtain from the apostles the gift of working miracles, and that he had formed an alliance with demons when his offers were repulsed."

For the first part of this statement there is the distinct authority of Justin Martyr, who, in his *Prima Apologia*, describes the statue with minuteness, and cites the inscription. But M. Figuier does not mention that, so lately as 1662, the following inscription existed in the island of the Tiber—SEMONI SANCO DEO FIDIO SACRUM; and that this has given rise to the supposition that Justin mistook a statue to Semo Sancus, the Sabine Deus Fidius, for one to his contemporary the magician.

Among the acts of Simon that have been recorded for the instruction of posterity, there is one that may be recommended to the notice of modern mediums. He did not turn tables, or cause furniture to dance, but he ordered a scythe to work by itself, and it performed an allotted task in a manner not to be surpassed by the most dexterous mower.

Of Apollonius it is said that he was able to transport himself instantaneously from one end of the earth to another, to change at pleasure into a bird, a stone, or a tree, to predict future events, and to evoke the spirits of the departed.

Besides Simon and Apollonius, the Roman dominions swarmed with less distinguished professors of the magic art; and the writings both of heathen philosophers and of Christian fathers contain numerous descriptions of their practices. M. Figuier quotes *Tertullian* and *Ammianus Marcellinus* to show that prophetic chairs and tables, circles of people, and divining perdules, were among the machinery employed, and then passes on to glance at the Alexandrian philosophy, which, after the suppression of the schools, became the heritage of so-called sorcerers. From this retrospect he draws the following conclusions:—

"What, then, were the means employed for the accomplishment of the various miracles to which we have so briefly referred? and how, among the ancients, did sorcerers proceed to work upon the rich mines of human weakness and credulity? This question, like all others which arise from facts dimly shadowed in the farthest distances of history, and having their origin in the most remote antiquity, cannot be resolved by any positive or documentary evidence. In default of such proofs there are, however, data which enable us to arrive, by induction, at probabilities that are little less than certain.

"An attentive examination of the chief marvels related in the histories of paganism, and of the earlier portion of the Christian era, shows that all these marvels might have been accomplished by a knowledge of certain physical principles and physical phenomena. This has been extremely well proved by a very ingenious and learned writer, who has devoted a part of his laborious life to historical researches into occult science. Eusèbe Salverte has shown, by a profound study of facts, and by inductive conclusions from them, that, in whatever time and place the occurrence of such prodigies has been recorded, there existed certain classes of philosophers who possessed scientific knowledge to a greater or less extent. In the skilful application of such rudimentary science, the priesthood found means to astonish, to alarm, and consequently to govern the vulgar.

"'Putting aside,' says Eusèbe Salverte, 'that which belongs to trickery, to imposture, or to hallucination, there are no ancient miracles which a man versed in modern science cannot reproduce, either immediately, or after applying himself to penetrate the mystery; and the same science gives facilities to work various other miracles, neither less brilliant nor less numerous than those which fill the pages of history. The example of what the moderns could effect in magic is sufficient to explain the magic of the ancients.'

"It is certain that modern physical science gives us the means of repeating the miracles of the ancients. To affirm, therefore, that knowledge of such science was required for their first performance is to advance more than a conjecture,—it is to form an induction that has almost the force of an inevitable conclusion."

M. Figuier proceeds to describe the gradual dispersion of the pagan priesthood and their neophytes before the advance of Christianity, and to assign to this cause the diffusion over the whole Roman empire of persons and practices once confined to the temples and holy places of mythology. Among the Druids, especially, the professors of the magic art found a refuge and a welcome; and when Christianity penetrated into Gaul, the Druid priesthood were discovered to be in the habit of commanding spirits and of exorcising demons. The like gifts claimed by the Christians furnished an occasion of bitter rivalry between the two religions, and the Christians, for the more effectual destruction of their adversaries, revived that argument which the founder of Christianity had condemned in the mouths of the pharisees. A demon expelled by a Christian priest, they said, was expelled by the mighty power of God; and the expulsion was a miracle. A demon expelled by a heathen priest was expelled by the aid of demons; and the expulsion was a sorcery. By virtue of this distinction the heathen priesthood were ranked with sorcerers and magicians, and under such denominations they were exiled, drowned, or burnt.

The very miracles which the Church had employed for the destruction of rival creeds without, were found useful, in the next place, for the settlement of doctrinal points within her bosom; and the same prodigies which, by their occurrence among the heathen, condemned those heathens as allies and servants of the devil, were held to be ranged upon the side of truth in all disputes between the Church and her own children. In case of schism, therefore, or of division into two religious parties, miracles were freely appealed to as the apostolic sign and the seal of tradition; and the Church, torn by many such divisions, and continually struggling against heresies, had constant necessity that such miracles should be worked. In order that the power of her priests over the devil should not be diminished by want of exercise, she had an equal necessity for demoniacs, and she found no lack of them. The Church distinguished—it is useless to inquire how—but she distinguished, between the persons who, by virtue of an agreement with the devil, had placed themselves voluntarily under his dominion, in consideration of being gifted by him with certain powers of infernal magic; and those whom the devil had seized upon by violence, or who were possessed through the intermediate agency of sorcerers. In all times, the first class of demoniacs were found to be extremely numerous; and it is impossible to say how many thousands of these unfortunates perished in the flames. Such was the rage to exorcise and to burn, that the monks discovered demoniacs wherever they had need of miracles, either to display the omnipotence of God, or to supply the tables of their convents. Unhappy, indeed, was he who was attacked by any malady. The most trivial indisposition might often be the cause of a horrible death, thanks to the zeal of the spiritual physicians who were eager for its cure.

From this time forwards, until the eighteenth century, the prevailing belief in the marvellous took the form of demonomania; and Figuier's pages contain little more than a catalogue of judicial murders. During the sixteenth and seventeenth centuries, a belief in the power of sorcerers was universal throughout Europe. The presence and the action of the devil in the human body was regarded as a familiar fact, altogether beyond dispute. When an individual was said to be possessed, no discussion was likely to arise about the actual fact of possession, but only as to whether it was brought about by the direct action of the devil or by the intermediate agency of a magician. Every sick person, for whose symptoms or sufferings the science of that time could neither assign a cause nor discover a remedy, was said to be possessed, and this convenient doctrine relieved the physicians and the moralists of the period from difficulty and embarrassing researches. An invalid suffering from convulsions, from any nervous disease, from any of the various hysterical or hypochondriacal affections, or from others which were imperfectly understood, was considered, by the mass of the people as well as by the educated classes, to be possessed by the devil.

It was the custom to refer to the devil himself, that is to say to the individual possessed, for information as to the manner in which the possession had been produced. This practice was extremely dangerous to many persons, inasmuch as the individual designated by the energumene as the author of the calamity could not be saved by any human agency; but, even if of the highest rank, or if an ecclesiastic, would nevertheless be proceeded against with the utmost rigour.

The Catholic Church provided a ritual for exorcisms, in which the following were enumerated as the signs or proofs of possession :—

1. Power to discover the unexpressed thoughts of the exorcised.
2. Knowledge by the possessed of foreign languages which he had not learned; and power to speak them.
3. Knowledge of future events.
4. Knowledge of events occurring at distant places, or beyond the reach of ordinary vision.
5. Sudden exaltation of the intellectual powers.
6. A development of physical strength greater than would properly belong to the age or sex of the possessed.
7. Suspension of the body of the possessed in the air during a considerable time.

The method in which these tests were applied in practice appears to have been somewhat similar to the manner in which the pretensions of a modern clairvoyant, or medium, are examined by a circle of enthusiasts. The curious love for the marvellous and the horrible, which is so marked a characteristic of the human mind, was sufficient to overpower anything like judicial impartiality, and to render the very judges themselves accomplices in pious frauds, intended to prove either the reality of the possession, or the guilt of the accused. In all cases, the confessions of the accused were accepted as sufficient and damning evidence against them, however they might be without corroboration, however much opposed to known facts, however plainly the offspring of frenzy or insane delusion. The practice of judging the so-called witches and sorcerers seems, indeed, to have developed an insanity all its own; and to have been the source of a delusion to the effect that the mass of mankind were in league with the devil. M. Figuier quotes from a personage whom he calls "le fameux Boguet," Chief Justice of the territory of St. Claude, who wrote in the reign of Henri IV.:—

"I hold," writes Boguet, "that the sorcerers could form an army equal to that of Xerxes, notwithstanding that it consisted of eighteen hundred thousand men. For Trois Echelles, one of the best inquirers into their affairs, declared, in the reign of Charles IX., that there were three hundred thousand in France alone; and how shall we calculate the number to be found in the remaining countries of the world? Shall we not believe that from them at least as many more might be collected? For my own part, I make no doubt that, if we glance only at our neighbours, we shall find them all alike, swarming with these miserable and accursed vermin. Germany can attend to nothing but the preparation of fires; Switzerland, for this cause, has depopulated many of her villages; Lorraine shows to travellers thousands upon thousands of stakes to which sorcerers have been bound; and for ourselves (we being no more exempt than others) we see the frequent executions that take place in many districts. Savoy sends us daily an infinite number of persons possessed by demons, who, being conjured, say that they were placed in the bodies of these poor creatures by sorcerers, and that most of those whom we have burned, here in Burgundy, came originally from thence. And what opinion shall we form regarding France? It is difficult, indeed, to believe that she can be cleansed, considering the number that she contained in the time of Trois Echelles; and, without taking more distant regions into account, the sorcerers walk about by thousands, multiplying on the earth like the caterpillars in our gardens. I would have them to know that, if I had my pleasure, the world would be cleansed thoroughly; for I should desire that they might all be united in one body—so as to be burned at one time in a single fire."

The benevolent intentions of this mediæval hero, although not absolutely accomplished, were not absolutely thwarted. For some twenty pages, the work of M. Figuier consists of nothing but a catalogue of judicial murders committed, not upon individuals, but upon masses of people. It is recorded that a single official, Nicolas Rémy, who exercised judicial authority in Lorraine towards the end of the sixteenth century, sent nine hundred reputed sorcerers, within fifteen years, to the stake or the gallows; and, nearly a hundred years later, the Parliament of Rouen addressed a solemn remonstrance to Louis XIV., who had designed to exert his prerogative of mercy in favour of some wretches sentenced to the stake, and had commuted their punishment to perpetual exile. The Parliament, composed of the most distinguished men in the province, commenced their memorial by reciting that the crime of sorcery had always been punished by death, in accordance with Scripture and with the testimony of the Fathers of the Church, by all the kings in Christendom. They proceeded to cite instances showing that successive Parliaments had invariably assigned the same penalty to the offence; and they appealed to the piety of the monarch for the maintenance of the customary rigour. "In 1675," writes M. Figuier, "when the Parliament of Normandy unanimously signed this remonstrance to the King, the *Misanthrope* and *Tartuffe* had already appeared upon the stage, and more than thirty years had elapsed since the foundation of the French Academy!"

The introductory and more general portion of the History of the Marvellous embraces a glance at the progress and effects of the belief in demoniacal possession from the death of Joan of Arc to the end of the seventeenth century. In order more minutely to describe the methods of procedure in cases of presumed sorcery, M. Figuier then enters upon a detailed narrative of the so-called possession of the Ursuline nuns at Loudun, in 1632, and the following years, and of the trial, condemnation, and execution of Urban Grandier, charged with having bewitched them. The history is one that cannot be profitably condensed, and that space does not permit us to extract; but it may be studied with advantage by those who would learn what kind of hysterical excitement was then believed to indicate possession, and what kinds of testimony were then (as now) accepted in proof of the various matters alleged. The next division of M. Figuier's work, under the title of *Les Convulsionnaires Jansénistes*, contains an account of the life and death of François de Paris, of the so-called miracles worked at his tomb in the cemetery of St. Medard, and of the various flagellations, crucifixions, and other torture, undergone by the fanatic devotees whose minds were excited upon the subject of his sanctity. This episode brings down the History of the Marvellous to the year 1787.

The second volume contains the Divining Rod and the Protestant Prophets.

M. Figuier traces the history of the divining rod in its three chief applications—for the detection of thieves, of minerals, and of springs of water—from the earliest period down to the researches of M. Chevreul, in 1812. M. Chevreul, by a series of careful experiments upon the suspended ring—the analogue of the divining rod—showed clearly that the movements of all such agents were produced by unconscious muscular action on the part of the persons holding them; the muscular action, in its turn, being governed by *expectant attention*, or an idea of the results likely to be produced. M. Chevreul's experiments and conclusions are detailed in the *Revue des Deux Mondes* for May, 1833; and it is unnecessary, in this place, to recapitulate matter that is, in its principles, so familiar to every physiologist. M. Figuier does not, however, mention (although the fact curiously illustrates the vitality of all such delusions) the resuscitation of the pendulum in late years, under the name of Rütter's magnetoscope, as a test of the veritable presence, in homœopathic globules, of the medicaments professedly contained in them; nor the experiments of Dr. Madden, of Torquay, who, following in the steps of M. Chevreul, showed that the oscillations of the magnetoscope were entirely governed by the mind of the operator, and corresponded with his own belief in the nature of the globules under examination. It is melancholy to reflect that these experiments should have been required after the publication of those by M. Chevreul, and at a time when the pretensions of the magnetoscope ought not to have excited a moment's attention on the part of persons ordinarily well informed.

The Protestant prophets, or Camisards, have been claimed as allies by some spiritualists of the present day. About 1668, the Protestants of France, maddened by the sanguinary persecutions that followed the revocation of the Edict of Nantes, deprived of their property, their civil rights, and often of their lives by the gibbet or the stake; unable to marry or to legitimize their offspring; inadmissible as witnesses; precluded from holding any office, or from following any professional or commercial calling, were driven into a frenzy bordering upon despair. Forced by persecution to fly from France, the pastors had said to their flocks—"Fear nothing; although we are forced to leave you, the Spirit of the Lord will not forsake you, but will be always present in your assemblies, and will speak to you through the mouths of women and children."

Understood literally, these comforting words had disordered the imagination of many of the unfortunates who were detained by poverty in their native country. Their churches being destroyed, and their worship forbidden, they assembled among woods and rocks for secret prayer. For these persecuted religionists, the mountains and deserts were peopled with phantoms, and filled with the voices of revelation. In a profound silence, the slightest sound was regarded as the word of the Holy Ghost; and, if we may believe Catholic writers, certain artifices were used to exhibit miraculous visions to the more simple—visions, which, in the opinion of the chiefs of the party, were likely to attract adherents to their cause. The armed insurrection that followed, and that continued, with various success, until its final suppression in 1705, was accompanied in its whole course by phenomena of ecstasy and of pretended inspiration. The prophets ruled the camp, and were chosen as military leaders on account of their spiritual gifts.

From M. Figuier's details, and copious extracts from contemporary documents, we gather that the supernatural manifestations among the Camisards were remarkably uniform in their character. With exceptions, and especially with exceptions in the case of those men whose talents and ambition gave them the desire and the capacity to rule their fellows, and in whom the means necessary to that end may pardonably be regarded with suspicion, the great majority of the inspired were women and young children, likely subjects for hysterical somnambulism. After the sermon of some desert preacher, or any other circumstance that had strongly fixed the thoughts upon religious ideas and upon the sufferings of the Church, the individual about to be inspired would remain for a time absorbed in his own reflections, and, wrapped in profound reverie, would become the subject of intense cerebral exaltation. After a longer or shorter duration of this phase, he would suddenly fall, deprived of feeling; and, stretched at length, would be attacked by epileptiform convulsions. By-and-bye, the scene gradually changed. The convulsions diminished and finally disappeared, quiet and serenity took the place of trembling and of pain. At length, the individual would rise apparently restored to himself, and would commence an eloquent discourse, preaching the truths of the Calvinist faith, denouncing the idolatry of the Papists, and prophesying future events, among which were always the destruction of the modern Babylon (Rome), and the restoration of the ruined churches. These discourses, which lasted sometimes for hours, were always in French, although the *Langue d'Oc* alone was spoken in all the provinces of the south. The first words were invariably these:—"I tell you, my child, I assure you, my child." It was the Holy Ghost who spoke thus by the mouth of the inspired. The oration being concluded, gradually, and by a scarcely appreciable transition, the prophet returned to his natural state and his ordinary language; not remembering, or remembering but confusedly, the words which he had spoken during the strange and temporary exaltation of his intellectual faculties.

Sometimes the ecstasy was provoked by the breathing of a prophet. In the religious meetings, the preacher, having concluded his discourse, would approach the neophytes considered worthy to receive the prophetic gift, and, breathing into the mouths of one or two, would say, "Receive the Holy Ghost." Soon after the newly elect would fall, would undergo tremblings and convulsions, would presently arise, and speak as if inspired. Having finished, he would in his turn breathe upon some other candidate for the like gifts, who, after his own period of excitement, would render the same service to others.

Moreover, a whole congregation, composed of perhaps a thousand persons, would sometimes fall into convulsions at the mere command of the preacher. Having finished his sermon, the prophet would exclaim, "*Mercy!*" loudly and repeatedly, and would order his flock to fall upon the ground. The greater number of the faithful would be obedient to this command.

The foregoing paragraphs (slightly condensed in translation), if stripped of a few unimportant particulars relating to time, place, and the accident of persecution, might serve for a description of much that occurred during the late revival at Belfast. They might serve also, M. Figuier remarks, to describe a similar movement in Sweden, in 1846; and all the phenomena appear to be purely, and in the truest sense, hysterical. The use of the French language is rendered less surprising by the sameness of the discourses; and the ignorance and stupidity of those who became eloquent when "inspired" is only an additional illustration of a very familiar fact. We have so lately discussed the Belfast revival, that this subject need not detain us longer.

The third volume of the History is devoted to "animal magnetism," from the first appearance of Mesmer before the public, to the latest applications of hypnotism; and may be divided into two portions, the first, a very graphic and entertaining narrative of events, the second, their physiological explanation. Under the latter head, M. Figuier disposes in succession of the various hypotheses that have been advanced by the ignorant and the credulous; and claims for hypnotism the power to explain all the phenomena of cataleptic trance, ecstasy, or somnambulism, and all the events that have supported, either in ancient or modern times, a belief in possession or in inspiration (excepting, of course, the inspiration made known to us by the evidence of Scripture). He admits, and regrets, that in the present state of knowledge, it is not possible to go much farther than this, or to state the essential changes in which hypnotism or nervous sleep consists.

We know only that it can be readily produced by expectant attention, and that such attention can be most easily commanded by the aid of some object on which to fix the senses and the thoughts. *How* the act of attention modifies the condition of the nervous system we have yet to learn; but we are certain that the influence is only self-contained, and independent of any external agency —whether of "spirit" or "fluid." That our knowledge extends no farther is due to two causes; first, to the discovery of anæsthesia, produced by chemical agents, a discovery that destroyed at once the surgical interest attaching to mesmerism; and, secondly, to the character of the persons by whom mesmerism was chiefly practised, and of the performances in which its effects were displayed. Respectable and intelligent practitioners were unwilling to investigate a pursuit that afforded notoriety to the most credulous of the profession, and gain to the most ignorant and fraudulent of tricksters. Now that the popular enthusiasm with regard to mesmerism has died away, now that clairvoyance and phreno-mesmerism are forgotten delusions—succeeded by the "spiritualism" of the day, there is a prospect of scientific inquiry into the basis of reality underlying all these things, and into the essential nature of the agencies concerned in producing them.

In the meanwhile, the phenomena which can be referred to hypnotism, or nervous sleep, may be stated as follows:—

1. Bodily insensibility, ranging in degree from that of natural sleep to the most complete unconsciousness of pain, or other external impressions.

2. Somnambulism, or a state of general unconsciousness, accompanied by an exaltation of sensibility with regard to impressions of a particular class. These may be indebted for their power, either to their nature or their source; that is to say, they may appeal to the sensorium by reason of being in harmony with the dominant idea, or by reason of emanating from a particular person; the attention being fixed subjectively upon an idea, in the first class, and objectively, upon an individual, in the second. The former condition may be illustrated by ecstasy and mesmeric clairvoyance, the latter by electro-biology; and in each there will be complete subjection of the belief and the will to the impressions conveyed through the senses. Thus, in ordinary mesmeric sleep-waking, or in the ecstasy of religious revivals, or during the exorcisms recorded by M. Figuier, the "subjects" were most acutely alive to every breath of suggestion from without that had reference to the matter actually in hand, hearing the faintest whispers, catching at the slightest hints; but neglecting, or even absolutely deaf and blind to, any irrelevant speech or action. The "electro-biologized" person, too, has his whole attention engrossed by the speech of the operator; and his powers of comparison, judgment, and volition paralysed—not by any agency from without, but because his nervous force is concentrated upon a single object. To these varieties of somnambulism, distinct or blended, differing in degree and duration under different circumstances, the greater portion of the phenomena called supernatural may be referred.

3. Catalepsy, or rigidity of the limbs in a position not determined by gravity, is, when universal, of comparatively rare occurrence. In a partial form, it probably is more common, plays its part in the maintenance of the attitudes of faquirism, and is familiar to the *habitués* of the mesmeric *séance*.

4. Convulsions and tremblings are ordinary forerunners of the complete development of any of the former states.

5. Muscular movements, involuntary and unconscious, are very frequent in cases of partial hypnotism, and are brought about by a degree of attention that would be insufficient to produce any of the former classes of phenomena. They are exemplified in table-turning, in the movements of the divining rod, the exploratory pendule, and the magnetoscope.

For all these there are at least two distinct sources; one, the contemplation of an emotion, or state of feeling, as instanced in all the varieties of ordinary hysteria; the other, the contemplation of an object, as in all the varieties of artificial hypnotism. In both, the operation of the state of attention upon the body is not limited to the muscular and nervous systems; but may extend, under certain circumstances, to every function of organic life.

We proceed now to the consideration of the matter that most immediately concerns us; that is to say, the relation borne by modern spiritualism to these by-gone marvels. We may remark, in the first place, that a popular belief in the agency of spirits as the active causes of supernatural phenomena, has alternated with a belief in the agency of *fluids*, variously described as electric, magnetic, or odylic. While, therefore, we are not entitled to speak of spiritualism as being, in its essential characters, a new doctrine, still we cannot but recognise the existence of novel elements in its modern phases; and we think it will be found that for these novel elements, for everything that is distinctive in its characteristics or teaching, it is, in a great degree, indebted to the impulse given to human thought by the writings of Emanuel Swedenborg.

Into the theological and doctrinal teaching of Swedenborg it is not our purpose to enter; but it is necessary to say a few words concerning his account of the eternal world or state, for the information of readers who may not be conversant with his views.

He held, then, that the soul, or spiritual existence, rather than the material body, constituted the essential and actual man; and that this soul, although not palpable or visible by material organs, was just as really a substance as the earthly body itself. He held that the spirit, when liberated from the body by the death of the latter, passed at once into an intermediate state of existence, the Hades of the Greek Scriptures, where it was prepared for its final destination, in heaven or in hell; and further, that, at certain epochs of the world's history, Hades had been cleared of its tenants by the operation of general judgments. Of these he taught that the final one, the last judgment predicted in the New Testament, occurred in or about the year 1757; and that, from that date for ever, individual judgments will do away with the occasion for general ones; each departed spirit going to his own place speedily. The last general judgment but one, according to this writer, took place during our Lord's ministry upon earth: so that for 1700 years or thereabouts, Hades had been continually receiving the spirits of those who departed this life, and of whom there were many completely assimilated to the state of the infernals, and ready to enter into Gehenna. He believed, also, that the spirits in Hades were able to communicate with men in this world, by suggesting thoughts to them—thoughts good or evil; and that they were actually and continually so employed; and he traced much of the profligacy and misery of the time in which he lived to the number of wicked spirits who were by this kind of suggestion constantly influencing the thoughts and actions of the human race; then, as at all former times, too ready to fall before temptation. As the immediate result of the last general judgment, and the removal, consequent upon it, of spirits confirmed in evil from Hades to Gehenna, he predicted a great amelioration in the condition of the human race; who, no longer brutalized by overpowering demoniac influences, were to make prodigious advances both in knowledge and in conduct. Moreover, these advances were to be permanent: because the individual judgments to be recorded for the future, and the more speedy transmission of souls in consequence of them through Hades to their final destination, would forbid any accumulation of demoniac influence in a state able to operate upon mankind.

Swedenborg taught, farther, that the spiritual world was around us on every side, and only concealed from our view by the veil of fleshly consciousness—a veil capable of being raised, prior to dissolution, whenever it so pleased Omnipotence. Its being thus raised was, he held, the means by which various persons described in Scripture—*e.g.*, Balaam and Elijah's servant—were enabled to behold spiritual existences formerly invisible to them; and he ascribed his own visions, into the nature of which we do not intend to enter, to a similar interposition of the Divine.

Now an absolute belief in Swedenborg's doctrines of Christian faith and duty, and a belief, more or less modified, in his visions, and his views of the eternal world and state, have for many years been very widely diffused among Protestant Christians, especially in the United States. The small and most sectarian sect called Swedenborgians, represent only a minute fraction of the followers of their master; and hence there were, when spirit-rapping was first heard of, thousands of people perfectly familiar with the idea of the nearness of the spirit world, and who believed that an alteration in their own state would, even in this life, allow them to hold actual and conscious communion with the souls of the departed.

It may here be observed, however, that while regarding spiritualism as in one sense a result of Swedenborg's doctrines, we do not wish to be understood as implying that it is their corollary. For these doctrines may be assented to by those who, nevertheless, feel that intercourse with the spirit world is something entirely abnormal; possible, it is true, and perhaps occasionally permitted; but that, nevertheless, the barriers between the natural and the spiritual states are not of a nature to be removed by any power short of Omnipotence, or to be suspended for the fulfilment of any purposes short of those of universal wisdom. The doctrine of the eternal spiritual existence of the human race is so completely in unison with human hopes and aspirations, is so agreeable to our reason, and so abundantly confirmed by revelation, that Swedenborg can add nothing to the evidence in its favour. His works should rather be regarded as a natural and necessary reaction against that Protestantism which, desiring to overthrow the Romish scheme of purgatory, had, in fact, utterly ignored the spiritual life of Hades, and had been gradually developed into a belief in the suspended animation of human souls, from the dissolution of the body to some period still in the future. Under this teaching, the soul or spirit, destined only to be resuscitated after the lapse of ages, ceased to be regarded with the interest properly attaching to it, and, instead of being considered the essential man, was looked upon as a vague and shadowy something of which it was impossible to give any precise account. The most enlightened Protestants of the eighteenth century, as a rule, would hold about the soul opinions only to be described as a fasciculus of negations; and would look forward to its reunion with the material body as a condition essential to its active life. The writings of Swedenborg, to minds thus instructed, would possess all the charm and interest of absolute and most attractive novelty; and, instead of only modifying erroneous opinions with regard to the state and condition of the soul after death, would in many cases present for the first time a real idea of the soul as a substantial existence. This idea thus given, and spiritual bodies being described as perpetually surrounding us, it is but a step farther to think of them as exerting active influences upon material things.

Accordingly, when, nearly ten years ago, it was announced that the then familiar table-turning was a result of spiritual forces, and that spirits communicated with mankind through the agency of persons called "mediums," by making raps upon tables, there were thousands in the middle and upper classes, of the kind called "intelligent observers," who saw no difficulty at all in the matter. They knew of no reason why these things should not be; they were conscious of no broadly-marked distinction between spirit and matter; they saw no incongruity in the presumed action and reaction between them; they heard the raps, and took the spirits for granted. From this, the delusion prospered; and, having at first been little more than a new method of drawing-room fortune-telling, it now numbers, in the United States by millions, and in this country probably by thousands, adherents who consult their familiar for every principle of conduct in this life, and for every belief or hope with regard to the life to come. Of this sect, the mediums are the priests, and raps upon a table are the oracles.

The pretensions of the Spiritualists have steadily increased during late years; but their present claims are stated as follows, by a prominent brother of the order, who wishes to "present a brief general statement of the leading phenomenal phases in which, at the present day, Spiritualism is presented to us:"—

"Before doing so," writes this author (*Spiritual Magazine*, No. 2), "as a preliminary observation necessary to a right understanding of the matter, we would remark that there are persons in some way peculiarly constituted whose presence appears to furnish conditions requisite to enable spirits to act upon matter, or to manifest their agency in any way cognizable to men. In what this peculiarity consists, whether it be chemical, electrical, magnetic, odylic, or in some combination of these, or in what else, it would lead us too far from our present purpose to consider." (We break into the quotation at this point, in order to call attention to the effects of spiritual intercourse upon English composition.) "At present we would only point out the fact that the presence of one such person at least is necessary in every circle before any spiritual manifestations can be obtained. Such persons are now technically designated *mediums*."

The most common form of the manifestations, and that which is most easily obtained, is seen in—

"1. *The Rappings, Table-tippings, and other Sounds and Movements of Ponderable Bodies.*—The company assembled place their hands lightly on a table, and, if a suitable medium is present, in a short time sounds, like raps or detonations are heard on the table, the chairs, the walls, or the floor, often varying in power and tone. . . . . At other times, instead of sounds being heard, extraordinary movements of the table are seen, it rising and falling vertically or perpendicularly, and to different elevations off the floor, or sliding along the room first in one direction and then in another, or moving rapidly round it . . . . On more than one occasion we have seen the table rise from the floor without any contact . . . . no one being nearer than from two to three feet of it. Human beings also have frequently been raised off the floor and floated round the room in the presence of numerous persons."

2. *Spirit-writings and Spirit-drawings.* — The former of these modes of communication is not unfrequent. Usually, the medium holds a pencil in hand as for writing, and, sometimes immediately, sometimes after a few minutes, the hand goes into involuntary motion, forming letters, words, and sentences, making an intelligible communication or reply to some question, verbal or mental, that has been asked. . . . . With some mediums the hand is simply used mechanically, the medium not having the slightest idea of what is being written; with others this is accompanied by impression as to the immediate word or sentence that is to be written, but no further. I know one medium who sees before him in the air, or upon the table, the word he has to write. . . . . Cases of direct spirit writing, that is, not requiring the intervention of a mortal hand, are comparatively rare.

3. *Trance and Trance-speaking.* . . . . . — In this state the trancee frequently speaks as from a spirit—sometimes in long and sustained discourse; and even at times in a foreign and (to the trancee) unknown tongue. We have scores of times heard persons of but little education discourse, when in this state, with an amplitude of knowledge which we are sure they did not in themselves possess, and with a logical coherence and power of expression of which in their normal state they were incapable. . . . . This state is similar, if not identical, with that which in the same persons may be induced by mesmerism.

4. *Clairvoyance and Clairaudience.* . . .

5. *Luminous Phenomena* are sometimes seen at spiritual *séances.* They are usually described as very brilliant; sometimes they appear as stars, or as balls of fire; at other times they shoot, meteor-like, through the apartment, or gleam over the walls, or appear as luminous currents circling round a particular centre, such as the hand of the medium, the pencil with which he is writing, or some object in the room.

6. *Spiritual Impersonation,* or the representation or reproduction in a medium of the actions and manner, gait, deportment, and other peculiarities which distinguished the actuating spirit in his earth-life.

7. *Spirit-music.*—A musical instrument, say a harp or an accordion, being held or suspended in the hand of the medium, or of some person near him, tunes are sometimes played on it by invisible agency, often in a very superior manner; sometimes it will be a known and familiar tune, at other times spirit-music will be thus improvised.

We know persons who often, when alone and unexpectedly, hear delightful music, apparently in the air, resembling, and yet unlike, any other they have heard. . . .

8. *Visible* and *Tactual Manifestations*, such as the appearance and touch of *spirit hands*. . . .

9. *Spirit Intercourse by means of the mirror, crystal, and vessel of water*. . . .

10. *Apparitions of the Departed*. . . .

11. *Visions and Pre-visions*.

12. *Dreams*. . . .

13. *Presentiments*. . . .

14. *Spirit Influx*, by which ideas and sentiments are infused into the mind. . . .

15. *Involuntary Utterance*. . . .

16. *Possession*.—We believe that many persons treated as insane are only so in the same sense as the demoniacs of old. . . .

Our space has compelled us entirely to omit the explanation of some of these sixteen headings; but we trust that we have preserved enough to convey to the reader a clear notion of the phenomena that are said to occur. In the fifth number of the *Spiritual Magazine* we find a paper by William Howitt, entitled "The Threefold Development of Spiritualism," from which we learn that there are three distinct phases of mediumship. Of these, the first, or lowest, is concerned in the production of the physical phenomena, the second involves intellectual, and the third, spiritual illumination. The poems of Mr. Harris, said to be dictations from the spirits of Rousseau, Keats, Shelley, Byron, Coleridge, Pollok, &c., are quoted as illustrations of the intellectual phase, and the sermons of Mr. Harris as illustrations of the spiritual phase. To these compositions we shall have occasion to refer hereafter.

Mr. Harris himself defines modern spiritualism "as a series of actions on and in the human spirit and body, and on the objects of the natural world, produced by the more abundant descent of the Divine Spirit into Christendom and the world, for the purpose of unfolding the more interior and spiritual, as well as natural human faculties, into higher states of force, perception, and utility. It may be defined, in its counter movement, as the results produced in man and on nature by the opposite effort of infernal spirits, to take advantage of new openings, to invite to evils, and to destroy the faith."

We have in these various quotations a sufficient basis of information concerning the alleged facts of spiritualism to enable us to investigate their nature and causes. The three phases of Mr. Howitt's are too much for us; and we purpose to consider the whole matter under two heads—first, the physical phenomena of a *séance*; and, secondly, the results of spiritual dictation.

(*To be continued.*)

